# Plasma free amino acid profiles are associated with serum high molecular weight adiponectin levels in Japanese medical check-up population without type 2 diabetes mellitus

**DOI:** 10.1007/s00726-023-03257-6

**Published:** 2023-03-17

**Authors:** Kengo Tokunaga, Hidehiro Nakamura, Sakino Toue, Yumiko Kato, Yosuke Ida, Sawako Miyoshi, Rika Yoneyama, Hiroaki Ohnishi, Tadakazu Hisamatsu, Susumu Okamoto

**Affiliations:** 1 Department of General Medicine, Kyorin University School of Medicine, 6-20-2 Shinkawa, Mitaka-shi, Tokyo, 181-8611 Japan; 2 Research Institute for Bioscience Products & Fine Chemicals, Ajinomoto Co., Inc, 1-1 Suzuki-cho, Kawasaki-ku, Kawasaki-shi, 210-8681 Japan; 3 Clinical Laboratory, Kyorin University Hospital, Mitaka-shi, Tokyo, Japan; 4 Department of Gastroenterology and Hepatology, Kyorin University School of Medicine, Mitaka-shi, Tokyo, Japan

**Keywords:** Plasma free amino acid profile, Adiponectin, Medical check-up, Type 2 diabetes mellitus

## Abstract

To prevent the progression of type 2 diabetes mellitus (T2DM), early detection and intervention are important. Several studies have already shown that the serum adiponectin level could be useful for evaluating the future risk of T2DM. Recently, plasma free amino acid (PFAA) concentrations have also emerged as potential biomarkers that predict the future onset of T2DM. In this study, we aimed to further characterise PFAA profiles by elucidating the association with the serum high molecular weight (HMW) adiponectin level in this cross-sectional study. A total of 1000 Japanese subjects who underwent medical check-ups were enrolled, and their plasma concentrations of 21 amino acids and clinical parameters were measured. The subjects without T2DM were divided into quartiles (Q1-4) by serum HMW adiponectin level, and the association with between PFAA concentrations was analysed. Concentrations of glutamate, alanine, proline, tyrosine, histidine, methionine, lysine, branched-chain amino acids (BCAAs) and tryptophan varied significantly according to the adiponectin quartile. Furthermore, serum adiponectin levels showed significant inverse correlations with these amino acids. The change in the PFAA profile in the group with the lowest adiponectin concentrations (Q1) was similar to that of T2DM patients. Although both adiponectin levels and PFAA concentrations are known to be altered by the accumulation of visceral fat and insulin resistance, the levels of glutamate, BCAA, lysine and tryptophan remain significantly associated with adiponectin level after adjustment for age, body mass index and homeostasis model assessment of insulin resistance, showing the direct association between PFAA concentrations and the serum HMW adiponectin level. ***Registration number*: **University Hospital Medical Information Network Clinical Trials Registry (UMIN-CTR) UMIN000029920, registered on Nov 13th 2017 (prospectively registered).

**Supplementary Information:**

The online version contains supplementary material available at 10.1007/s00726-023-03257-6.

## Introduction

Metabolic syndrome, which is mainly caused by the accumulation of visceral fat, is a pathophysiological state that causes type 2 diabetes mellitus (T2DM). T2DM has become a major health issue worldwide, including in Asian regions, since constant high blood glucose levels can damage both the large and small blood vessels of various organs, which leads to macrovascular/microvascular complications, including cardiovascular disease, ischaemic heart disease and dysfunction in the eyes, kidneys, feet and nerves (Orasanu and Plutzky 2009). These health problems have a negative impact on not only patients’ quality of life but also medical costs; they require continuous clinical care and management that consume significant health-care resources. To prevent T2DM and the progression of these complications, it is critically important to detect the risk in the early phase of metabolic syndrome and for patients to change their lifestyles.

Previous studies have found that adipose tissue is an endocrine organ that produces and secretes various bioactive substances, referred to as adipocytokines (Scherer et al. 1995; Maeda et al. 1996). Adiponectin, an adipocytokine, is known to work as an insulin sensitiser and modify glucose homeostasis (Kadowaki et al. 2006). The concentration of adiponectin in blood shows a strong inverse association with the progression of insulin resistance and T2DM (Ziemke and Mantzoros 2010; Li et al. 2009). Furthermore, some previous longitudinal studies have shown that the circulating adiponectin level predicts subsequent changes in insulin resistance and the incidence of T2DM within several years (Choi et al. 2004; Yamamoto et al. 2004), suggesting that the adiponectin level could be useful as a biomarker that reflects the future risk of T2DM onset.

Several recent studies have shown that plasma free amino acid (PFAA) profiles are altered prior to the development of T2DM or cardiovascular events. In prospective cohort studies such as the Framingham Offspring Study, PFAA levels, particularly the levels of branched-chain amino acids (BCAAs) and aromatic amino acids, were significantly associated with a future diagnosis of T2DM (Wang et al. 2011) and cardiovascular diseases (Magnusson et al. 2013). Other studies also revealed that several amino acids were significantly altered in Japanese people with high visceral obesity independent of their body mass index (BMI) and that a specific formula incorporating some PFAA concentrations correlated with visceral fat area was able to predict the four-year risk of developing new-onset lifestyle-related diseases, including T2DM, metabolic syndrome and dyslipidaemia (Yamakado et al. 2015). All these data suggest that PFAAs could be another novel potential biomarker for predicting the future risk of T2DM and other lifestyle-related diseases.

Although these previous reports suggest that both adiponectin and PFAAs could be useful for the early detection of future T2DM risk, the potential of PFAAs as biomarkers has not been fully elucidated compared with that of the serum adiponectin level, which has been widely regarded as a surrogate marker for metabolic status in the body. To the best of our knowledge, few studies have investigated the relationship between adiponectin and PFAAs. A previous study showed that the levels of several amino acids were highly correlated with adiponectin and insulin-related markers in T2DM patients (Nakamura et al. 2014). Another study reported a significant correlation of the high molecular weight (HMW) adiponectin level with amino acid levels, but this observation is limited for BCAAs (Katagiri et al. 2018). In this cross-sectional study, we investigated the alteration of PFAA profiles, i.e., 21 amino acids, according to circulating HMW adiponectin levels and clarified their association in a large healthy Japanese population undergoing health check-ups at our hospital. Through this analysis, we attempted to characterise PFAA profiles as potential biomarkers.

## Materials and methods

### Ethics statement

This study was conducted in accordance with the Declaration of Helsinki, and the protocol was approved by the Faculty of Medicine Research Ethics Committee at Kyorin University and the ethics committees of Ajinomoto Co., Inc. All the subjects gave their written informed consent for inclusion before they participated in the study. All the data were analysed anonymously throughout the study. The study was registered in the University Hospital Medical Information Network Clinical Trials Registry (UMIN-CTR) UMIN000029920.

### Subjects

This study was a cross-sectional evaluation of the relationship between the adiponectin level and levels of glucose-, insulin- and lipid-related variables obtained with clinical laboratory tests. From December 2017 to March 2019, a total of 1000 individuals utilised the Ningen Dock comprehensive medical check-up system at Kyorin University Hospital and gave their informed consent for participation in this study.

### Measurement of metabolic variables and quantification of PFAAs

Blood samples were taken from the subjects after overnight (> 8 h) fasting. Fasting plasma glucose (FPG) was measured with the hexokinase method, and haemoglobin A1c (HbA1c) and serum HMW adiponectin were quantified using the latex agglutination immunoassay. Serum insulin levels were measured immunologically. Serum concentrations of triglycerides were measured using an enzymatic colorimetric assay (Sekisui Medical, Tokyo, Japan), and low-density lipoprotein (LDL) cholesterol and high-density lipoprotein (HDL) cholesterol were measured using direct methods (Sekisui Medical). All clinical laboratory tests were performed by SRL (Tokyo, Japan). The homeostasis model assessment of insulin resistance (HOMA-IR) was calculated using the following equation: HOMA-IR = Fasting insulin level (μU ml^−1^) × Fasting plasma glucose level (mg dl^−1^)/405.

For the amino acid analyses, blood samples (5 ml) were collected from a forearm vein into tubes containing disodium ethylenediaminetetraacetate after overnight fasting and were immediately placed on ice. The plasma was isolated by centrifugation and then stored at − 80 °C until analysis. The plasma samples were deproteinised with acetonitrile at a final concentration of 80% before measurement. The plasma amino acid concentrations were measured by high-performance liquid chromatography–electrospray ionization mass spectrometry followed by precolumn derivatization as previously described (Shimbo et al. 2009a, 2009b). The following 21 amino acids were measured: alpha aminobutyric acid (aABA), alanine (Ala), arginine (Arg), asparagine (Asn), citrulline (Cit), glutamine (Gln), glutamate (Glu), glycine (Gly), histidine (His), isoleucine (Ile), leucine (Leu), lysine (Lys), methionine (Met), ornithine (Orn), phenylalanine (Phe), proline (Pro), serine (Ser), threonine (Thr), tryptophan (Trp), tyrosine (Tyr), and valine (Val).

### Statistical analysis

Among the 1000 participants, those who had missing data (*n* = 2) and/or had been diagnosed with type 2 diabetes (medical history and/or FPG ≥ 126 mg/dL, HbA1c ≥ 6.5%) (*n* = 96) were excluded. Participants were divided into quartiles according to HMW adiponectin levels (Q1-4). Differences in participant characteristics among the quartiles were tested using analysis of variance for continuous variables. For multivariate analysis, the adiponectin level and HOMA-IR were log-transformed. Statistical and multivariate analyses were performed with the JMP 12.2.0 program (SAS Institute Inc., Cary, NC, USA). The data in the tables are expressed as the mean ± SD. The datasets generated during and/or analysed during the current study are not publicly available due to ethical restrictions but are available from the corresponding author on reasonable request.

## Result

The subjects without T2DM were classified according to their serum HMW adiponectin quartiles. Since sex is also known to be a strong factor that influences both plasma amino acid concentrations and serum adiponectin levels, we also stratified them by sex. Table 1 shows the subject’s basic characteristics, including diabetes-related markers. As previous studies have indicated (Katagiri et al. 2018), subjects in the lowest HMW adiponectin quartile (Q1) showed higher BMI, HOMA-IR, and triglyceride values and lower HDL cholesterol values for both men and women. On the other hand, there were no associations between the adiponectin quartiles and blood glucose-related parameters such as FPG and HbA1c. Subjects with T2DM showed remarkably higher HOMA-IR, FPG and HbA1c values for both sexes.

**Table 1:** Basic characteristics of subjects according to their HMW adiponectin level quartile

Adiponectin quartile (μg/mL)	Men						Women					
	Q1 (< 1.8)	Q2 (1.8–2.7)	Q3 (2.7–4.0)	Q4 (4 ≤)	*P* value	T2DM (3.5 ± 2.3)	Q1 (< 4.0)	Q2 (4–5.7)	Q3 (5.7–7.7)	Q4 (7.7 ≤)	*P* value	T2DM (4.0 ± 1.8)
N	138	149	138	150		79	80	80	84	83		17
Age (years)	56.5 ± 9.9	58.2 ± 10.4	59.2 ± 11.2	61.5 ± 9.9	**0.0005**	64.8 ± 9.0	56.9 ± 10.9	59.5 ± 10.9	56.8 ± 9.5	60.4 ± 9.6	0.0538	63.5 ± 8.3
BMI (kg/m^2^)	25.5 ± 3.3	24.5 ± 3.0	24.3 ± 3.1	22.4 ± 2.4	** < 0.0001**	24.4 ± 3.6	22.5 ± 3.0	22.2 ± 2.8	21.2 ± 2.6	20.3 ± 2.8	** < 0.0001**	26.6 ± 5.7
HOMA-IR	2.0 ± 1.3	1.6 ± 0.9	1.3 ± 0.9	1.1 ± 0.7	** < 0.0001**	3.5 ± 7.0	1.5 ± 1.0	1.2 ± 0.7	1.0 ± 0.6	1.0 ± 0.5	** < 0.0001**	6.6 ± 8.9
FPG (mg/dL)	102.9 ± 9.2	102.8 ± 8.8	102.5 ± 11.4	102.0 ± 9.8	0.8363	140.9 ± 35.9	97.7 ± 10.5	98.2 ± 8.8	96.0 ± 9.0	96.6 ± 8.6	0.4080	158.7 ± 60.0
HbA1c (%)	5.8 ± 0.3	5.8 ± 0.3	5.7 ± 0.3	5.8 ± 0.3	0.6762	7.1 ± 1.0	5.8 ± 0.4	5.8 ± 0.3	5.7 ± 0.3	5.7 ± 0.3	0.1886	7.6 ± 1.5
HDL cholesterol (mg/dL)	49.8 ± 11.8	53.8 ± 13.2	60.1 ± 14.8	67.6 ± 14.9	** < 0.0001**	56.0 ± 12.6	64.2 ± 13.7	69.6 ± 14.7	73.6 ± 15.8	82.0 ± 15.3	** < 0.0001**	67.3 ± 12.1
LDL cholesterol (mg/dL)	121.4 ± 31.5	124.4 ± 28.0	121.5 ± 30.5	117.0 ± 30.5	0.2042	105.2 ± 28.3	124.1 ± 29.3	127.1 ± 28.7	115.4 ± 26.5	118.6 ± 29.5	**0.0401**	123.4 ± 33.5
Triglyceride (mg/dL)	153.5 ± 91.1	135.6 ± 76.3	130.2 ± 97.3	90.4 ± 47.4	** < 0.0001**	121.2 ± 67.7	102.7 ± 49.8	89.3 ± 36.1	76.5 ± 36.7	69.0 ± 26.6	** < 0.0001**	107.5 ± 56.4
Total protein (g/dL)	7.1 ± 0.3	7.1 ± 0.4	7.1 ± 0.4	7.1 ± 0.4	0.2718	7.2 ± 0.4	7.1 ± 0.4	7.1 ± 0.3	7.0 ± 0.3	7.0 ± 0.3	0.5844	7.2 ± 0.4
Albumin (g/dL)	4.5 ± 0.2	4.4 ± 0.3	4.4 ± 0.3	4.4 ± 0.3	0.0703	4.4 ± 0.3	4.4 ± 0.2	4.3 ± 0.2	4.4 ± 0.2	4.3 ± 0.3	0.1447	4.4 ± 0.3

Values are shown as mean ± SD. Significant differences among quartiles were evaluated using analysis of variance and highlighted in bold letters

Table 2 shows PFAA profiles according to HMW adiponectin quartiles. Concentrations of Glu, Ala, Pro, Tyr, His, Met, Lys, Val, Leu, Ile and Trp varied significantly according to the adiponectin quartiles in both men and women. Concentrations of all these amino acids in the lowest adiponectin quartile (Q1) were the highest in the all quartiles, although women in the Q2 group showed the highest values of Ala, Pro, His and Lys. Among men, Gly and Cit values were the lowest in the Q1 group.

**Table 2:** Plasma amino acid concentrations according to the HMW adiponectin level quartile

Adiponectin quartile	Men						Women					
	Q1 (< 1.8)	Q2 (1.8–2.7)	Q3 (2.7–4.0)	Q4 (4 ≤)	*P* value	T2DM (3.5 ± 2.3)	Q1 (< 4.0)	Q2 (4–5.7)	Q3 (5.7–7.7)	Q4 (7.7 ≤)	*P* value	T2DM (4.0 ± 1.8)
Glu	56.6 ± 19.6	49.6 ± 19.1	43.8 ± 17.4	35.9 ± 13.9	** < 0.0001**	51.1 ± 20.6	40.2 ± 15.9	34.4 ± 14.5	30.3 ± 13.0	29.6 ± 12.8	** < 0.0001**	47.9 ± 19.6
Ser	109.1 ± 16.1	106.9 ± 17.3	106.6 ± 17.8	110.5 ± 17.8	0.1639	114.0 ± 18.5	111.0 ± 21.7	113.8 ± 18.8	117.2 ± 18.8	116.8 ± 17.6	0.1324	111.6 ± 17.5
Asn	46.8 ± 5.8	46.5 ± 6.0	46.2 ± 6.2	47.4 ± 6.5	0.3506	47.0 ± 6.3	43.5 ± 5.9	45.6 ± 6.0	44.3 ± 6.2	44.0 ± 6.6	0.1766	42.9 ± 5.0
Gly	196.3 ± 39.3	199.5 ± 39.0	199.9 ± 38.1	209.4 ± 37.5	**0.0243**	205.8 ± 48.5	225.8 ± 58.8	234.8 ± 63.4	238.5 ± 62.3	240.6 ± 57.6	0.4213	224.9 ± 70.2
Gln	571.6 ± 60.8	581.4 ± 64.3	579.2 ± 60.4	594.7 ± 64.6	**0.0171**	575.4 ± 64.7	551.0 ± 55.9	574.0 ± 55.7	562.4 ± 61.9	561.1 ± 64.2	0.1159	548.8 ± 70.7
His	83.5 ± 7.9	85.1 ± 8.1	82.9 ± 9.0	80.7 ± 8.2	** < 0.0001**	83.5 ± 8.5	78.5 ± 7.2	80.5 ± 7.5	78.3 ± 7.9	77.2 ± 7.3	**0.0448**	75.9 ± 7.9
Thr	125.9 ± 22.6	127.2 ± 22.1	126.5 ± 22.7	123.1 ± 22.1	0.4063	125.4 ± 25.1	116.9 ± 24.8	120.9 ± 25.6	117.3 ± 26.3	111.0 ± 24.8	0.0968	116.3 ± 27.0
Ala	378.8 ± 85.0	363.2 ± 65.8	348.2 ± 77.7	326.2 ± 75.2	** < 0.0001**	368.2 ± 73.0	322.4 ± 64.7	323.2 ± 54.3	300.1 ± 64.2	285.4 ± 71.4	**0.0002**	343.9 ± 70.0
Cit	31.4 ± 7.2	32.0 ± 6.0	32.0 ± 5.8	34.0 ± 7.3	**0.0033**	34.7 ± 9.5	31.1 ± 6.5	32.1 ± 7.7	31.2 ± 6.6	33.1 ± 6.9	0.2199	31.3 ± 10.0
Arg	94.9 ± 14.0	94.6 ± 14.5	92.7 ± 14.6	93.7 ± 16.7	0.5971	95.2 ± 15.9	88.8 ± 15.8	90.3 ± 14.3	86.3 ± 16.0	86.2 ± 16.4	0.2526	84.7 ± 14.4
Pro	162.2 ± 37.8	151.6 ± 34.2	147.7 ± 45.0	138.2 ± 39.4	** < 0.0001**	156.6 ± 41.7	129.8 ± 34.0	140.6 ± 48.8	125.0 ± 40.6	111.1 ± 26.0	** < 0.0001**	154.6 ± 47.1
a-ABA	22.6 ± 7.0	22.4 ± 6.5	22.5 ± 6.5	20.9 ± 6.6	0.1056	25.4 ± 10.3	20.3 ± 5.6	20.9 ± 6.0	20.6 ± 5.3	18.6 ± 5.6	**0.0399**	22.5 ± 6.9
Tyr	68.9 ± 13.1	67.0 ± 10.2	68.0 ± 12.2	63.8 ± 10.6	**0.0010**	67.3 ± 11.9	61.8 ± 10.0	61.1 ± 8.7	59.2 ± 8.8	57.9 ± 7.8	**0.0199**	68.4 ± 13.9
Val	263.3 ± 40.1	248.4 ± 34.0	237.2 ± 27.9	223.8 ± 33.6	** < 0.0001**	261.5 ± 54.2	210.4 ± 30.9	206.9 ± 31.9	194.4 ± 25.5	183.3 ± 24.1	** < 0.0001**	237.7 ± 39.3
Met	28.0 ± 4.1	27.4 ± 4.9	27.0 ± 3.9	26.0 ± 3.5	**0.0004**	27.7 ± 4.8	24.2 ± 3.5	24.0 ± 3.1	23.8 ± 3.1	22.6 ± 2.7	**0.0063**	24.7 ± 3.1
Orn	53.7 ± 10.3	52.4 ± 10.2	51.1 ± 10.2	54.1 ± 11.9	0.0773	60.4 ± 15.4	49.7 ± 11.0	50.4 ± 12.8	45.6 ± 10.0	47.0 ± 12.6	**0.0292**	56.4 ± 24.0
Lys	203.0 ± 25.6	202.0 ± 27.4	196.9 ± 26.1	192.2 ± 29.2	**0.0022**	202.3 ± 25.6	182.1 ± 27.3	187.4 ± 28.1	175.9 ± 24.7	170.4 ± 23.9	**0.0003**	193.8 ± 26.2
Ile	77.9 ± 14.1	71.8 ± 12.2	68.4 ± 11.2	63.8 ± 11.6	** < 0.0001**	77.6 ± 20.6	56.4 ± 8.9	54.4 ± 11.8	50.5 ± 7.6	47.1 ± 6.6	** < 0.0001**	63.5 ± 13.7
Leu	147.1 ± 21.7	138.6 ± 19.9	132.1 ± 17.8	123.1 ± 19.0	** < 0.0001**	145.7 ± 29.5	111.9 ± 13.0	109.2 ± 19.5	102.2 ± 11.9	96.5 ± 11.0	** < 0.0001**	125.0 ± 23.8
Phe	63.5 ± 9.0	62.5 ± 7.6	62.6 ± 8.4	59.6 ± 7.5	**0.0002**	64.0 ± 7.6	57.0 ± 5.5	56.1 ± 6.9	55.6 ± 5.7	56.3 ± 7.7	0.6030	61.9 ± 8.7
Trp	62.2 ± 8.0	61.3 ± 8.9	60.1 ± 8.3	57.7 ± 7.6	** < 0.0001**	58.9 ± 8.1	55.8 ± 8.5	54.6 ± 5.8	53.9 ± 7.5	51.8 ± 7.8	**0.0058**	52.8 ± 5.2

Values are shown as mean ± SD (μmol/L). Significant differences among quartiles were evaluated using analysis of variance and highlighted in bold letters

**Fig. 1:**
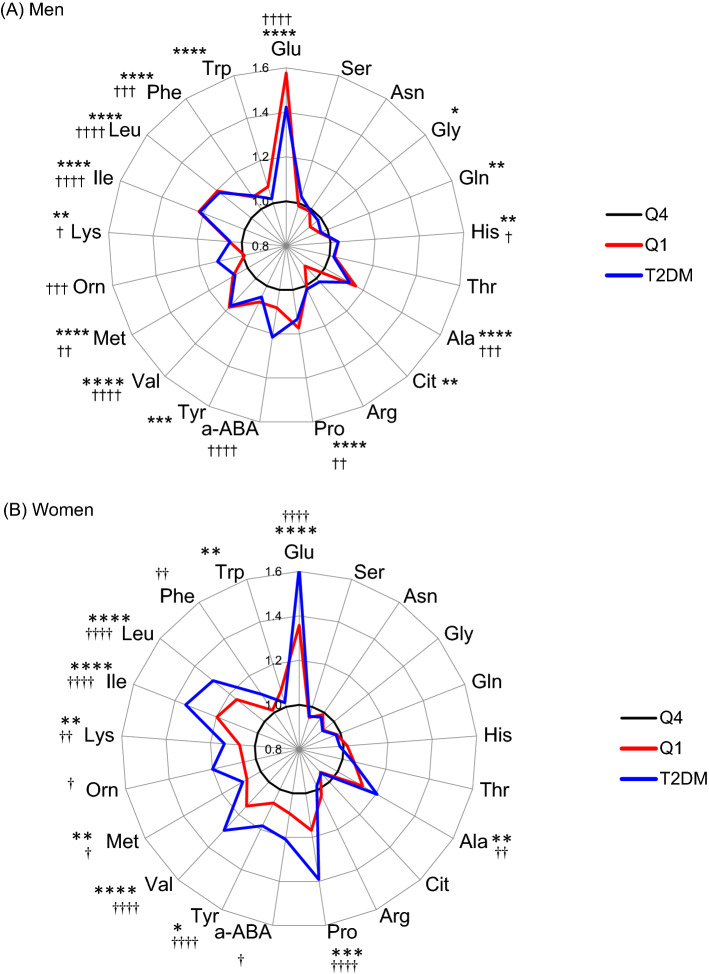
Alterations in PFAA concentrations in the lowest HMW adiponectin quartile and T2DM patients. Radar charts show relative values of 21 plasma amino acid concentrations in the lowest HMW adiponectin quartile (Q1) (red line) and T2DM patients (blue line) compared with those in the highest HMW adiponectin quartile (Q4) for men (**A**) and women (**B**). Statistical differences were evaluated by ANOVA followed by Dunnett’s test. *P < 0.05, **P < 0.01, ***P < 0.001 and ****P < 0.0001 between Q1 and Q4. †*P* < 0.05, ††*P* < 0.01, †††*P* < 0.001 and ††††*P* < 0.0001 between T2DM and Q4

The radar charts in Fig. 1 show the relative value of each PFAA among subjects in the lowest adiponectin quartile (Q1) and in T2DM subjects compared with those in the highest adiponectin quartile (Q4). Concentrations of Glu, Ala, Pro, Tyr, Val, Met, Lys Ile, Leu and Trp in Q1 were significantly higher than those in Q4; in particular, Glu, Pro and BCAA levels were increased by 20–60% and 10–40% in men and women, respectively. The PFAA alterations in the Q1 quartile showed a similar pattern to those in T2DM for both men and women, although aABA and Orn levels were significantly increased only in T2DM patients.

Pearson’s correlation coefficients between PFAA levels and clinical variables for non-T2DM subjects are shown in Table 3. Consistent with the results of Table 2, the HMW adiponectin level showed significant negative correlations with BMI, HOMA-IR and various PFAAs. Among the PFAAs, the adiponectin level was significantly correlated with Glu (*r* = − 0.4126 and − 0.3268 for men and women, respectively), Val (*r* = − 0.4035 and − 0.3318), Ile (*r* = − 0.3899 and − 0.3614) and Leu (*r* = − 0.4259 and − 0.3674). Although these amino acids were also significantly associated with HOMA-IR, there were no or weak correlations with FPG and HbA1c. Similar relationships were also observed for men with T2DM; Glu and BCAA levels were significantly and inversely associated with adiponectin (supplementary table 1).

**Table 3:** Pearson’s correlation coefficients between blood glucose-related parameters and plasma amino acid concentrations

	Men					Women				
	Adiponectin	BMI	HOMA-IR	FPG	HbA1c	Adiponectin	BMI	HOMA-IR	FPG	HbA1c
Adiponectin	–	– 0.3621****	– 0.3739****	– 0.0629	– 0.0653	–	– 0.3028****	– 0.2750****	– 0.0818	– 0.1544**
BMI	– 0.3621****	–	0.4998****	0.0928*	0.1714****	– 0.3028****	–	0.5341****	0.2827****	0.2833****
HOMA-IR	– 0.3739****	0.4998****	–	0.4255****	0.2423****	– 0.2750****	0.5341****	–	0.5527****	0.2713****
FPG	– 0.0629	0.0928*	0.4255****	–	0.4810****	– 0.0818	0.2827****	0.5527****	–	0.4724****
HbA1c	– 0.0653	0.1714****	0.2423****	0.4810****	–	– 0.1544**	0.2833****	0.2713****	0.4724****	–
Glu	– 0.4126****	0.3865****	0.4849****	0.2709****	0.1471***	– 0.3268****	0.3895****	0.3764****	0.3208****	0.1384*
Ser	0.0362	– 0.0601	– 0.0925*	– 0.0054	– 0.0213	0.1210*	– 0.1050	– 0.2478****	– 0.2075***	– 0.1668**
Asn	0.0137	– 0.0987*	– 0.2117****	– 0.0570	0.0331	0.0328	– 0.2747****	– 0.2435****	– 0.1012	– 0.0715
Gly	0.1366**	– 0.1221**	– 0.1524***	– 0.0993*	– 0.0055	0.0691	0.0287	– 0.0719	– 0.1448**	– 0.0959
Gln	0.1256**	– 0.0226	– 0.0972*	– 0.0772	0.1620****	0.0855	– 0.0217	– 0.1120*	0.0245	0.1596**
His	– 0.1514***	0.1188**	0.0522	0.0058	– 0.0071	– 0.0405	0.1039	– 0.0486	– 0.0593	0.0278
Thr	– 0.0675	0.1415***	0.0879*	0.0914*	0.0042	– 0.0574	0.0244	– 0.0986	– 0.1088*	– 0.0878
Ala	– 0.2876****	0.2788****	0.4215****	0.2668****	0.2003****	– 0.2121***	0.3091****	0.3315****	0.2321****	0.1639**
Cit	0.155***	– 0.1385***	– 0.1456***	– 0.0412	0.1186**	0.0984	– 0.0760	– 0.0622	0.0349	0.1367*
Arg	– 0.0494	– 0.0117	0.0316	0.0465	0.1964****	– 0.0402	– 0.0493	– 0.0617	0.1014	0.1957***
Pro	– 0.2109****	0.1569***	0.2695****	0.1067*	0.0860*	– 0.1953***	0.1079	0.1287*	0.0803	0.0380
a-ABA	– 0.0975*	0.0635	– 0.1132**	0.0071	– 0.0535	– 0.1310*	0.1901***	0.0138	– 0.0118	– 0.0265
Tyr	– 0.1775****	0.3282****	0.3719****	0.1954****	0.2339****	– 0.1596**	0.3134****	0.257****	0.2440****	0.1797**
Val	– 0.4035****	0.3518****	0.3439****	0.0785	0.1333**	– 0.3318****	0.4058****	0.3557****	0.2926****	0.2712****
Met	– 0.1962****	0.2410****	0.1992****	0.0439	0.1523***	– 0.1543**	0.0172	0.0083	– 0.0035	0.0058
Orn	0.0267	0.0798	0.1255**	0.0451	0.1812****	– 0.0937	0.1041	0.0924	0.1457**	0.2156****
Lys	– 0.1695****	0.1555***	0.1056*	0.0385	0.2240****	– 0.1447**	0.0668	0.0613	0.1945***	0.2313****
Ile	– 0.3899****	0.3757****	0.3427****	0.0567	0.1618****	– 0.3614****	0.3222****	0.3397****	0.2310****	0.1720**
Leu	– 0.4259****	0.3707****	0.2690****	0.0164	0.1367**	– 0.3674****	0.3549****	0.2806****	0.2096***	0.2185****
Phe	– 0.1791****	0.2777****	0.2508****	0.0679	0.2620****	– 0.0784	0.2100***	0.2087***	0.2241****	0.2309****
Trp	– 0.2005****	0.1600***	0.1725****	– 0.0082	0.1170**	– 0.1651**	0.0325	0.0065	0.0461	0.0902

Adiponectin and HOMA-IR values were log-transformed. Significant correlations were highlighted in bold letters

*P < 0.05

**P < 0.01

***P < 0.001

****P < 0.0001

Since significant correlations between PFAA concentrations and BMI or HOMA-IR were observed, PFAA concentrations were adjusted for age, BMI and HOMA-IR by multivariate analysis to remove their confounding effect on the relationship between the levels of adiponectin and PFAAs (Table 4). The results showed that significant associations between the adiponectin level and Glu, BCAA, Lys or Trp concentrations were still observed after adjustment for age, BMI and HOMA-IR for both men and women.

**Table 4:** Plasma amino acid concentrations according to the HMW adiponectin level quartile after adjustment for Age, BMI and HOMA-IR

	Men					Women				
	Q1	Q2	Q3	Q4	*P* value	Q1	Q2	Q3	Q4	*P* value
Glu	51.2 ± 17.3	47.7 ± 16.7	44.7 ± 16.0	41.8 ± 14.4	** < 0.0001**	37.5 ± 13.8	33.3 ± 12.8	31.4 ± 13.5	32.2 ± 11.7	**0.0160**
Ser	109.5 ± 16.2	107.1 ± 17.1	106.5 ± 17.2	110.1 ± 17.3	0.1965	111.9 ± 21.1	114.4 ± 17.7	115.6 ± 18.0	116.9 ± 16.6	0.3621
Asn	47.4 ± 5.9	46.7 ± 5.7	46.0 ± 6.0	46.8 ± 6.2	0.2842	44.1 ± 5.5	46.0 ± 5.5	43.9 ± 6.1	43.4 ± 6.2	**0.0315**
Gly	198.9 ± 40.2	200.5 ± 37.9	199.5 ± 36.8	206.5 ± 36.8	0.2956	226.3 ± 6.7	234.4 ± 6.7	237.0 ± 6.6	242.0 ± 6.6	0.4071
Gln	575.3 ± 60.4	582.9 ± 63.0	577.8 ± 61.2	591.0 ± 64.0	0.1466	555.9 ± 52.8	572.9 ± 53.3	562.2 ± 60.1	557.7 ± 64.4	0.2444
His	83.0 ± 7.9	85.0 ± 8.1	82.9 ± 9.0	81.3 ± 8.1	**0.0021**	78.4 ± 7.4	80.3 ± 7.4	78.1 ± 7.6	77.6 ± 7.3	0.1104
Thr	124.0 ± 22.3	126.7 ± 22.0	126.5 ± 22.2	125.2 ± 21.8	0.7294	116.5 ± 24.6	121.1 ± 24.3	116.0 ± 24.9	112.5 ± 25.1	0.1772
Ala	360.6 ± 75.6	356.5 ± 65.1	352.2 ± 74.1	345.8 ± 67.8	0.3188	312.7 ± 6.8	318.9 ± 6.8	305.1 ± 6.6	293.9 ± 6.7	0.0529
Cit	32.2 ± 7.0	32.3 ± 5.9	31.9 ± 5.3	33.1 ± 7.0	0.4181	31.8 ± 5.8	32.0 ± 7.3	31.4 ± 6.5	32.3 ± 6.5	0.8436
Arg	94.9 ± 14.0	94.5 ± 14.5	92.8 ± 14.5	93.7 ± 16.6	0.6746	89.9 ± 15.3	90.2 ± 14.4	86.4 ± 15.8	85.2 ± 15.5	0.0873
Pro	156.5 ± 35.2	149.4 ± 35.0	149.1 ± 45.0	144.2 ± 37.8	0.0619	126.3 ± 34.7	140.3 ± 48.0	125.5 ± 40.2	114.2 ± 24.5	**0.0003**
a-ABA	22.7 ± 6.8	22.5 ± 6.3	22.3 ± 6.4	20.9 ± 6.6	0.0728	20.2 ± 5.6	20.7 ± 5.9	20.6 ± 5.1	19.0 ± 5.5	0.1966
Tyr	66.6 ± 11.4	66.2 ± 9.9	68.3 ± 11.2	66.4 ± 9.8	0.2934	60.8 ± 8.9	60.4 ± 8.0	59.8 ± 8.3	58.9 ± 8.4	0.4890
Val	255.1 ± 37.0	245.7 ± 33.4	238.0 ± 28.1	233.2 ± 32.5	** < 0.0001**	205.9 ± 25.4	204.0 ± 31.5	197.1 ± 23.2	187.7 ± 23.3	** < 0.0001**
Met	27.5 ± 0.3	27.2 ± 0.3	27.0 ± 0.3	26.7 ± 0.3	0.4229	24.1 ± 3.6	24.0 ± 3.0	23.8 ± 3.0	22.7 ± 2.7	**0.0178**
Orn	53.4 ± 10.0	52.2 ± 9.6	51.2 ± 9.8	54.5 ± 11.8	**0.0446**	49.8 ± 1.2	49.8 ± 1.2	46.2 ± 1.2	46.8 ± 1.2	0.0603
Lys	201.8 ± 24.9	201.6 ± 26.2	196.8 ± 25.8	193.9 ± 28.7	**0.0268**	182.8 ± 2.8	186.4 ± 2.8	177.0 ± 2.7	169.6 ± 2.7	**0.0001**
Ile	74.9 ± 12.8	70.8 ± 11.7	68.6 ± 11.0	67.3 ± 11.5	** < 0.0001**	54.9 ± 8.0	53.7 ± 11.5	51.3 ± 7.4	48.5 ± 6.6	** < 0.0001**
Leu	142.8 ± 20.4	137.3 ± 19.3	132.2 ± 17.8	128.3 ± 18.7	** < 0.0001**	109.7 ± 11.2	108.0 ± 18.9	103.2 ± 11.9	98.8 ± 11.3	** < 0.0001**
Phe	62.4 ± 8.4	62.1 ± 7.4	62.7 ± 7.8	60.9 ± 7.2	0.2027	56.5 ± 4.9	55.7 ± 6.4	56.0 ± 5.8	56.7 ± 7.6	0.7329
Trp	61.3 ± 8.0	61.0 ± 9.1	60.2 ± 8.0	58.8 ± 7.4	**0.0422**	55.6 ± 8.7	54.7 ± 5.8	53.8 ± 7.4	52.1 ± 7.8	**0.0202**

Values are shown as mean ± SD (μmol/L). Significant differences among quartiles were evaluated using analysis of variance and highlighted in bold letters

## Discussion

In the current cross-sectional study, we investigated the association between serum HMW adiponectin levels and PFAA concentrations in a large number of Japanese people who underwent medical check-ups. The results showed that the HMW adiponectin level in nondiabetic subjects was significantly and inversely correlated with BCAA, Glu, Lys and Trp concentrations, and this association remained even after adjustment for age, BMI and HOMA-IR, suggesting that their relationship could be independent of fat mass or insulin resistance.

Although a previous study has already shown that plasma BCAA concentrations are significantly associated with adipokine levels, including both total and HMW adiponectin and leptin, in a Japanese population without diabetes (Maeda et al. 1996), to our knowledge, this is the first study to investigate their association with levels of 21 amino acids. Consistent with a previous report, our study also showed the strongest correlation among the level of each BCAA, HMW adiponectin levels and HOMA-IR, which is generally recognised as a marker of insulin resistance. To date, many studies have shown that plasma BCAA levels correlate with insulin resistance (White and Newgard 2019). One of the mechanisms for the elevation of plasma BCAA levels associated with insulin resistance was thought to be a decrease in BCAA catabolism. A decrease in both insulin action and utilization of amino acids in muscle causes a lower uptake of BCAAs into muscle (Pozefsky et al. 1969). In addition, subjects with insulin resistance showed decreased concentrations of branched-chain keto acids and α-ketoglutarate and increased acylcarnitine concentrations in muscle (Lerin et al. 2016), which suggests low activity of BCAA aminotransferase, the first enzyme to catalyse reversible transamination of all BCAAs to branched-chain keto acids. Other studies revealed that BCAAs are also metabolised in visceral adipose tissues, and insulin resistance causes a decrease in the expression levels of adipose tissue BCAA-catabolizing enzymes (Herman et al. 2010; Lackey et al. 2013). In our study, however, the association between the adiponectin level and BCAA concentrations was significant even after adjustment for BMI and HOMA-IR, suggesting a direct connection between them. Another study using mice showed that adiponectin rectifies elevated BCAA levels in blood induced by a high-fat diet (Liu et al. 2013; Lian et al. 2015), which suggests that alteration of BCAA concentrations might be directly reflected by a change in adiponectin level. Further investigation is needed to more precisely clarify the mechanism for the relationship among adiponectin, BCAAs and insulin resistance.

The plasma Glu concentration was also highly and negatively correlated with adiponectin levels. The Glu concentration in plasma is known to change drastically depending on the handling of blood samples. For instance, its concentration in whole blood left at room temperature for only 10 min is increased by more than 20% due to the action of glutaminase, which converts Gln into Glu (Takehana et al. 2016), meaning that strict sample management is required for the accurate quantification of Glu. In this study, we cooled whole blood samples immediately (i.e., less than 1 min) after blood collection, and this procedure enabled us to reveal the precise associations between the plasma Glu level and other variables. It has been reported that the Glu level in plasma is correlated more highly with visceral fat area than with subcutaneous fat area (Yamakado et al. 2012). Since the visceral fat area has been thought to be related to insulin resistance or decreased levels of adiponectin, our results are consistent with the report. A recent study has shown that the abundance of *Bacteroides thetaiotaomicron* in stool samples, a glutamate-fermenting commensal, is markedly decreased in obese individuals and is inversely correlated with the Glu concentration in serum. Furthermore, gavage with *B. thetaiotaomicron* reduced the plasma glutamate concentration and alleviated diet-induced body weight gain and adiposity in mice (Liu et al. 2017). The authors also reported that the relative abundance of *B. thetaiotaomicron* was positively correlated with circulating adiponectin levels and negatively correlated with circulating leptin levels. All these results suggest that the gut microbiome might play a central role in the mechanism underlying the strong relationship between Glu and adiponectin levels.

A significant change in Trp levels according to serum adiponectin quartiles was also observed. The Trp concentration in plasma has been known to be correlated with the status of protein nutrition, since Trp binds to albumin in blood. The current study also showed a significant correlation between Trp and albumin levels (data not shown). On the other hand, the albumin level was not changed among the adiponectin quartiles, suggesting that the relationship between Trp and adiponectin levels observed in the current study is not the result of a difference in protein nutrition. Although alteration of Trp levels caused by insulin resistance and T2DM is controversial (Oxenkrug 2015; Herrera et al. 2003; Chen et al. 2016), Trp metabolism is known to be highly associated with insulin resistance and T2DM (Le Floc’h et al. 2011; Oxenkrug 2013). The expression of indoleamine-2,3-dioxygenase (IDO), which is a rate-limiting enzyme of the Trp-kynurenine pathway, was enhanced, and metabolites in this pathway, such as kynurenine, were shown to be increased in obese people (Favennec et al. 2015). Since the kynurenine pathway and its metabolites are related to inflammation, further investigation will be needed to clarify the impact of inflammation associated with insulin resistance or adiponectin action on the plasma Trp level.

A previous study revealed a correlation between PFAA profiles (Glu, Ala, Trp and BCAAs) and adiponectin levels in T2DM patients (Nakamura et al. 2014). In the current study, we also obtained similar results: Glu and BCAA levels, which showed a significant correlation with the adiponectin level in non-T2DM subjects, were also negatively associated with adiponectin levels in T2DM men, although this result was not observed for T2DM women, probably due to the small number of subjects. This suggests that their correlation could be maintained independent of the onset of T2DM. Since T2DM patients might take antidiabetic drugs before the time of blood collection (the proportion is unclear in the present study), glucose-related parameters would not be suitable for monitoring the metabolic states in the body. PFAA concentrations and the adiponectin level could be potential indicators that cannot be estimated by circulating blood glucose levels independent of the onset of T2DM.

In the current study, subjects without T2DM in the lowest HMW adiponectin quartile (Q1) showed a similar change in the PFAA profile as that in T2DM subjects when compared with subjects in the Q4 quartile. Daimon et al. conducted 5-year follow-up examinations and showed that decreased serum adiponectin levels were an independent risk factor for the progression to T2DM in a Japanese population (Daimon et al. 2012), suggesting that subjects in Q1 were more likely to develop T2DM than those in Q4. Likewise, several studies have reported that changes in PFAA profiles occur before the onset of T2DM. The Framingham Offspring Study revealed that PFAA levels, particularly the levels of BCAAs and aromatic amino acids such as Phe and Tyr, were significantly associated with a future diagnosis of T2DM and cardiovascular diseases (Wang et al. 2011). In addition, a study that enrolled Japanese subjects showed that levels of several amino acids and a PFAA index (constructed to model the relationships between the PFAA profiles and the visceral fat area by multiple linear regression) showed good capabilities to predict future risk of developing new-onset lifestyle-related diseases. These results indicate that the PFAA profile and adiponectin level are potential biomarkers for predicting the future risk of T2DM. In fact, the technology has been applied to a risk screening test named “AminoIndex_TM_ Life Style Risk Screening” in Japan, which predicts the risk of developing T2DM within 4 years based on PFAA profiles (Nagao and Kimura 2020). Furthermore, both markers are known to be normalised by various types of interventions. Reduction of ~ 10–20% body weight in obese subjects or a specific diet, such as a low-fat diet, daily supplementation with fish or omega 3 fatty acids, and fibre supplementation, significantly increased the concentration of adiponectin in serum (Salas-Salvadó et al. 2006; Kasim-Karakas et al. 2006; He et al. 2004; Nelson et al. 2007; Micallef and Garg 2009; Grunberger et al. 2007). Weight loss induced by the diet and exercise intervention normalised the circulating levels of PFAAs (Tochikubo et al. 2016). These results suggest that both markers could be useful for not only detecting the risks of developing lifestyle-related diseases in the future but also monitoring improvements in physiological conditions.

The current study has several limitations. First, this is a cross-sectional study, which makes it difficult for us to confirm the direct or causal relationship between plasma amino acid profiles and the serum HMW adiponectin level. Although we adjusted it with some potential confounding factors such as BMI and HOMA-IR, the possibility that other confounding factors might exist cannot be eliminated. In addition, the value of BMI cannot provide accurate data for visceral adiposity which is more closely linked to metabolic syndrome, since BMI itself cannot differentiate between lean mass and fat mass. More suitable parameters should be taken into account for further analysis. Second, we did not assess dietary habits among subjects in this study. Indispensable amino acids including BCAAs, Lys and Trp cannot be synthesized in the body and their homeostasis partially depends on the intake from diet, which suggests that the amount of dietary intake of these amino acids could be reflected on their plasma concentrations. A previous study has shown that the contribution of lifestyle factors including dietary BCAA intake to plasma BCAA concentrations is much less than that of BMI (Hamaya et al. 2022), suggesting that dietary patterns would have little impact on our results. However, subjects enrolled in the previous study showed much higher BMI than those in this study conduted in Japan, which could make the contribution of BMI relatively larger. Further investigations will be needed to clarify the precise association between plasma amino acid profiles and the metabolic state in the body.

In conclusion, this study clarified the significant association between PFAA concentrations and the serum HMW adiponectin level. In addition to the serum adiponectin level which has been widely recognised as a useful biomarker for the detection of T2DM risk, PFAA concentrations could be used as a potential biomarker. Further longitudinal studies are needed to confirm and compare their performance as an efficient biomarker of the future T2DM risk and identify the underlying mechanism of this association.

## Supplementary Information

Below is the link to the electronic supplementary material.

Supplementary file1 (DOCX 34 KB)

## Data Availability

The datasets generated during and/or analysed during the current study are not publicly available due to ethical restrictions but are available from the corresponding author on reasonable request.
