# Supplementary material for: Plasma free amino acid profiles are associated with serum high molecular weight adiponectin levels in Japanese medical check-up population without type 2 diabetes mellitus
Source: Amino Acids. 2023 Mar 17;55(5):639–49. doi: 10.1007/s00726-023-03257-6 (PMC10247567; doi:10.1007/s00726-023-03257-6)
Supplement: Supplementary file 1 — Supplementary file1 (DOCX 34 KB) [file 726_2023_3257_MOESM1_ESM.docx]

**Supplementary table 1. Pearson’s correlation coefficients between blood glucose-related parameters and plasma amino acid concentrations in subjects with T2DM**

|  | Men with T2DM | | | | | | | | |  | Women with T2DM | | | | | | | | |
| --- | --- | --- | --- | --- | --- | --- | --- | --- | --- | --- | --- | --- | --- | --- | --- | --- | --- | --- | --- |
|  | Adiponectin |  | BMI |  | HOMA-IR |  | FPG |  | HbA1c |  | Adiponectin |  | BMI |  | HOMA-IR |  | FPG |  | HbA1c |
| Adiponectin | - |  | -0.2168 |  | -0.1888 |  | **-0.3438**** |  | **-0.3178**** |  | - |  | -0.2781 |  | **-0.5388*** |  | 0.3466 |  | -0.1612 |
| BMI | -0.2168 |  | - |  | **0.4325****** |  | **0.2867*** |  | 0.0979 |  | -0.2781 |  | - |  | 0.2453 |  | -0.1533 |  | 0.0543 |
| HOMA-IR | -0.1888 |  | **0.4325****** |  | - |  | **0.2866*** |  | 0.176 |  | **-0.5388*** |  | 0.2453 |  | - |  | 0.3763 |  | **0.5789*** |
| FPG | **-0.3438**** |  | **0.2867*** |  | **0.2866*** |  | - |  | **0.8519****** |  | 0.3466 |  | -0.1533 |  | 0.3763 |  | - |  | 0.4596 |
| HbA1c | **-0.3178**** |  | 0.0979 |  | 0.176 |  | **0.8519****** |  | - |  | -0.1612 |  | 0.0543 |  | **0.5789*** |  | 0.4596 |  | - |
| Glu | **-0.6165****** |  | **0.3055**** |  | **0.4179***** |  | **0.2483*** |  | 0.1972 |  | -0.3437 |  | 0.3676 |  | 0.0727 |  | -0.2384 |  | 0.203 |
| Ser | 0.123 |  | -0.1085 |  | -0.1442 |  | -0.0859 |  | 0.0033 |  | 0.4663 |  | 0.351 |  | -0.1668 |  | 0.2832 |  | 0.0051 |
| Asn | **0.3081**** |  | -0.1093 |  | -0.0722 |  | **-0.3442**** |  | **-0.2666*** |  | 0.4647 |  | -0.4752 |  | **-0.5167*** |  | -0.2553 |  | -0.2268 |
| Gly | **0.2550*** |  | -0.0047 |  | -0.2078 |  | **-0.3849***** |  | **-0.2875*** |  | -0.1849 |  | 0.2349 |  | 0.1823 |  | -0.4127 |  | -0.0136 |
| Gln | **0.4328****** |  | **-0.3043**** |  | **-0.2646*** |  | **-0.5562****** |  | **-0.3362**** |  | 0.2616 |  | -0.4546 |  | -0.4611 |  | -0.4395 |  | -0.4151 |
| His | 0.0486 |  | **0.2813*** |  | 0.1117 |  | -0.099 |  | -0.1189 |  | -0.197 |  | 0.3873 |  | -0.2124 |  | **-0.5546*** |  | -0.3227 |
| Thr | 0.0915 |  | 0.0743 |  | -0.091 |  | -0.1152 |  | -0.0803 |  | 0.1549 |  | -0.4045 |  | -0.3015 |  | -0.0587 |  | -0.2117 |
| Ala | -0.128 |  | 0.1192 |  | **0.2485*** |  | **0.3581**** |  | **0.3106**** |  | -0.4078 |  | 0.216 |  | 0.4076 |  | -0.0757 |  | 0.0156 |
| Cit | **0.3348**** |  | -0.1705 |  | -0.1828 |  | **-0.2769*** |  | -0.0968 |  | 0.2846 |  | **-0.6765**** |  | **-0.4851*** |  | -0.1692 |  | -0.1636 |
| Arg | 0.0083 |  | -0.1488 |  | -0.1836 |  | -0.1837 |  | 0.0835 |  | 0.1622 |  | -0.3586 |  | -0.3707 |  | -0.1085 |  | -0.3759 |
| Pro | -0.0814 |  | **0.3973***** |  | 0.2159 |  | -0.006 |  | -0.0822 |  | -0.244 |  | -0.1421 |  | 0.1188 |  | -0.1996 |  | -0.1937 |
| a-ABA | -0.0522 |  | -0.0249 |  | -0.086 |  | 0.1518 |  | -0.0364 |  | 0.4101 |  | 0.0914 |  | **-0.5193*** |  | -0.2081 |  | -0.1561 |
| Tyr | -0.0639 |  | 0.2187 |  | **0.2377*** |  | 0.1319 |  | 0.0826 |  | 0.0187 |  | -0.2319 |  | 0.2316 |  | **0.6723**** |  | -0.0019 |
| Val | **-0.4784****** |  | **0.2723*** |  | 0.1714 |  | 0.1399 |  | 0.0684 |  | -0.1041 |  | 0.2934 |  | 0.2512 |  | 0.29 |  | 0.0624 |
| Met | 0.1427 |  | 0.0177 |  | 0.053 |  | -0.074 |  | -0.0472 |  | 0.2163 |  | **-0.5074*** |  | -0.0997 |  | 0.1994 |  | 0.0053 |
| Orn | 0.0999 |  | -0.0203 |  | -0.0363 |  | **-0.3608**** |  | **-0.2596*** |  | **0.5256*** |  | -0.4466 |  | -0.103 |  | **0.5515*** |  | -0.2723 |
| Lys | **-0.4212***** |  | 0.1066 |  | -0.06 |  | 0.0658 |  | 0.1453 |  | 0.3359 |  | -0.4741 |  | -0.2532 |  | 0.0357 |  | 0.0206 |
| Ile | **-0.2627*** |  | 0.2118 |  | 0.1911 |  | 0.0909 |  | 0.0985 |  | -0.2565 |  | 0.4122 |  | 0.368 |  | 0.1981 |  | 0.1452 |
| Leu | **-0.3832***** |  | 0.2208 |  | 0.1139 |  | 0.1106 |  | 0.0933 |  | -0.307 |  | 0.4745 |  | 0.3563 |  | 0.1397 |  | 0.2121 |
| Phe | 0.0006 |  | 0.0577 |  | 0.2185 |  | 0.0269 |  | 0.0191 |  | -0.299 |  | -0.1747 |  | 0.1713 |  | -0.0229 |  | -0.0843 |
| Trp | -0.1665 |  | -0.1502 |  | -0.0621 |  | 0.0446 |  | 0.1848 |  | -0.3185 |  | -0.0975 |  | 0.0819 |  | -0.0517 |  | -0.2505 |

Adiponectin and HOMA-IR values were log-transformed. **P* <0.05, ***P* <0.01, ****P* <0.001, *****P* <0.0001.
